# High efficiency carbon nanotubes-based single-atom catalysts for nitrogen reduction

**DOI:** 10.1038/s41598-023-36945-0

**Published:** 2023-06-19

**Authors:** Wei Liu, Kai Guo, Yunhao Xie, Sitong Liu, Liang Chen, Jing Xu

**Affiliations:** 1 College of Optical, Mechanical and Electrical Engineering, Zhejiang A&F University, Hangzhou, 311300 Zhejiang People’s Republic of China; 2 School of Physical Science and Technology, Ningbo University, Ningbo, 315211 Zhejiang People’s Republic of China

**Keywords:** Catalysis, Theory and computation, Carbon nanotubes and fullerenes

## Abstract

Carbon-based single-atom catalysts (SACs) for electrochemical nitrogen reduction reaction (NRR) have received increasing attention due to their sustainable, efficient, and green advantages. However, at present, the research on carbon nanotubes (CNTs)-based NRR catalysts is very limited. In this paper, using FeN_3_@(n, 0) CNTs (n = 3 ~ 10) as the representative catalysts, we demonstrate that the CNT curvatures will affect the spin polarization of the catalytic active centers, the activation of the adsorbed N_2_ molecules and the Gibbs free energy barriers for the formation of the critical intermediates in the NRR processes, thus changing the catalytic performance of CNT-based catalysts. Zigzag (8, 0) CNT was taken as the optimal substrate, and twenty transition metal atoms (Sc, Ti, V, Cr, Mn, Fe, Co, Ni, Cu, Zn, Nb, Mo, Tc, Ru, Rh, Pd, W, Re, Ir, and Pt) were embedded into (8, 0) CNT via N_3_ group to construct the NRR catalysts. Their electrocatalytic performance for NRR were examined using DFT calculations, and TcN_3_@(8, 0) CNT was screened out as the best candidate with a low onset potential of − 0.53 V via the distal mechanism, which is superior to the molecules- or graphene-support Tc catalysts. Further electronic properties analysis shows that the high NRR performance of TcN_3_@(8, 0) CNT originates from the strong *d*-2π* interaction between the N_2_ molecule and Tc atom. TcN_3_@(8, 0) CNT also exhibits higher selectivity for NRR than the competing hydrogen evolution reaction (HER) process. The present work not only provides a promising catalyst for NRR, but also open up opportunities for further exploring of low-dimensional carbon-based high efficiency electrochemical NRR catalysts.

## Introduction

Ammonia (NH_3_) is not only essential for the production of fertilizers, but also a promising carbon-free energy carrier for hydrogen energy storage^1–3^. With the ever-increasing demand for NH_3_, the artificial synthesis of NH_3_ by nitrogen (N_2_) conversion has attracted extensive attention. Currently, industrial NH_3_ synthesis mainly relies on Haber–Bosch method, which commonly needs extreme reaction conditions (15–35 MPa and 400–600 °C)^4,5^ with massive energy consumption and simultaneously leads to amount of CO_2_ emission^6–8^. Hence, it is vital to develop a promising, efficient, and mild approach for sustainable production of NH_3_. Inspired by biological enzyme nitrogen fixation, electrocatalytic N_2_ reduction reaction (NRR) has been regarded as one of the promising NH_3_ production methods^9–11^. As the core component of electrocatalytic NRR system, a large number of electrocatalysts have been predicted through screening catalysts with different compositions and structures^12–16^, and some electrocatalysts also have been experimentally fabricated for NH_3_ production at ambient conditions^17,18^. However, due to the extremely high stability of N_2_ and high energy barrier for N≡N bond cleavage, exploring novel catalysts with high activity and low cost has always been an important goal and challenge of NRR research^5,19^.

As a class of novel heterogeneous catalysts, single atom catalysts (SACs) based on carbon materials have been successfully used for N_2_ fixation due to their lowly coordinated electronic structure and efficient utilization of the active species^13,14,20–22^. Up to now, various SACs based on graphene or nitrogen-doped graphene for NRR have been reported both experimentally and theoretically^17,23–26^, and the controllability of the coordination between metal atoms and substrate has also been realized experimently^26–28^. Additionally, one-dimension carbon nanotubes (CNTs) have also been used as SAC substrates due to their high surface area, high stability, high conductivity, and tailorable properties. CNT-based SACs have already emerged as promising electrocatalysts to catalyze oxygen reduction reactions^29–31^ and oxygen evolution reactions^32,33^, and the inherent properties of CNTs are believed to significantly contribute to enhancing the catalytic performance. The surface of CNT can be regarded as a curved graphene surface, which provides a potential means to use this feature to tune the catalytic performance. However, there are still relatively few researches on CNT-based SACs in the field of NRR^17,34^. Therefore, it is critical to investigate the structures, catalysis performance and reaction mechanisms of SACs based on CNTs to advance the development of highly efficient electrocatalysts for NRR.

In this work, using the density functional method, the effect of CNT curvatures on the catalytic performance of CNT based catalysts for NRR were investigated firstly. According to the criteria proposed for the screening of eligible electrocatalysts for NRR, zigzag (8, 0) CNT was selected as the optimal substrate to construct CNT based NRR catalysts. Through screening twenty transition metal atoms, Tc atom were found to exhibit the best N_2_ to NH_3_ conversion capabilities via the distal pathway with the extremely low limiting potential (− 0.53 V). Further calculations were performed to investigate the electronic properties to explain the high NRR performance of TcN_3_@(8, 0) CNT, and to evaluate the selectivity between NRR and hydrogen evolution reaction (HER).

## Computational details

All calculations are based on density functional theory as implemented in the Vienna ab initio simulation package (VASP)^35^. Our exchange-related functional adopts the revised Perdew–Burke–Ernzerhof (rPBE)^36^ under the generalized gradient approximation (GGA) method^37^. The projector augmented wave (PAW) method^38^ is used to describe the ion–electron interaction, and the PAW cutoff is set to 550 eV. The van der Waals interaction is calculated using the DFT-D3(IVDW = 11) method^39^. 1 × 1 × 3 supercells of carbon nanotubes are used to construct the structure models. In order to eliminate the interactions between two periodic repeating structures, the lattice parameters in the vacuum directions are set as 25.0 Å. The Brillouin zone is sampled using the Monkhorst–Pack k-point mesh^40^ of 1 × 1 × 2. All the structures are fully optimized until the energy convergence standard of 10^–5^ eV and the force convergence standard of − 0.01 eV/Å are reached. To further investigate the structural stability of the most likely carbon nanotube-supported monatomic catalysts, Ab initio molecular dynamics (AIMD) simulations in the canonical ensemble (NVT) with the Nose´–Hoover thermostat^41^ were performed at 500 K for 5 ps with a time-step of 1.0 fs.

The Gibbs free energy change (ΔG) in each elementary step is calculated based on the computational hydrogen electrode (CHE) model proposed by Norskov et al.^42^. The free energy of a proton–electron pair (H^+^ + *e*^−^) is equivalent to 1/2 H_2_ (g) under standard reaction conditions (pH = 0, 298.15 K, 101.325 kPa) at an external potential of 0 V. The free energy of the H* (ΔG_H_) is calculated to be − 0.62 eV in this work. The following equation is used for the calculations^43^:

1 $$\Delta {\text{G }} = \, \Delta {\text{E }} + \, \Delta {\text{ZPE }}{-}{\text{ T}}\Delta {\text{S }} + \, \Delta {\text{G}}_{{\text{U}}} + \, \Delta {\text{G}}_{{p{\text{H}}}} .$$

Among them, ΔE is the electron energy difference between two intermediates; ΔZPE is the change in zero point energy; T is the temperature (298.15 K); ΔS is the change in entropy calculated by frequency; ΔG_U_ = − *e*U, it represents the contribution of the electrode potential U to the free energy, where *e* is the number of transferred electrons, and U is the applied electrode potential; ΔG_*p*H_ = *k*_B_T × ln10 × *p*H, it represents the free energy correction of *p*H, where *k*_B_ is the Boltzmann constant, and *p*H value is set to be zero.

In addition, the adsorption energy is defined as:

2 $${\text{E}}_{{{\text{ads}}}} = {\text{ E}}_{{{\text{ads}} - {\text{sub}}}} - {\text{ E}}_{{{\text{ads}}}} - {\text{ E}}_{{{\text{sub}}}} ,$$

where E_ads-sub_ represents the total energy of the system after adsorption, and E_ads_ is the total energy of the adsorbent, E_sub_ represents the total energy of the substrate.

## Results and discussion

### Effect of CNT curvature on the catalytic performance of CNT based catalysts

**Figure 1:**
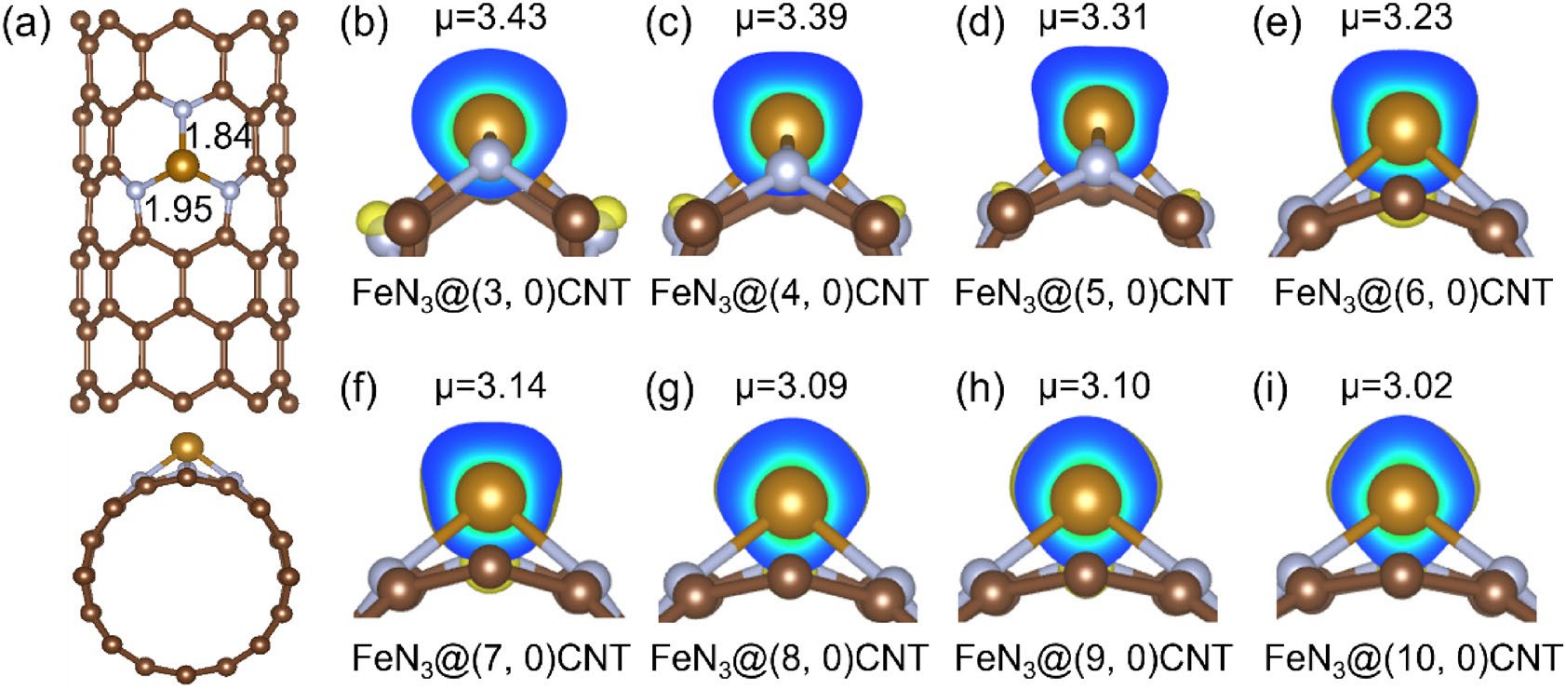
(**a**) Top and side views of the optimized structure of FeN_3_@(8, 0) CNT; (**b**–**i**) The spin-resolved density pictures of FeN_3_@(n, 0) CNTs (n = 3–10). The isovalue is set to be 0.015 eÅ^−2^, and the numbers are the spin magnetic moments of the Fe atoms.

Initially, eight zigzag (n, 0) CNTs (n = 3–10), were considered as substrates to anchor transition metal (TM) atoms to construct single atom catalysts. As shown in Fig. 1a, on the surface of a CNT, one carbon atom is deleted to form a single-vacancy defect, and then three carbon atoms possessing dangling bonds are substitutionally doped with nitrogen atoms to form a N_3_ group. The TM atoms are adsorbed at the center of the N_3_ groups. Previous studies have shown that FeN_3_-embedded graphene exhibits excellent catalytic performance for the N_2_-to-NH_3_ conversion^13^. Therefore, Fe was employed as the representative TM atom to study the effect of CNT curvature on the NRR catalytic performance of TMN_3_@(n, 0) CNTs.

The spin magnetic moment of the Fe center in FeN_3_@graphene is proved to be critical for the activation of the inert N_2_ molecule by the catalysts^13^. Therefore, the spin-resolved density of FeN_3_@(n, 0) CNTs were investigated firstly, and the results (Fig. 1b–i) demonstrate that all the FeN_3_ centers are highly spin-polarized. The Fe atoms protrude outside the CNT surfaces, and the charge clouds is distributed near the Fe atoms, indicating that the Fe atoms contribute most of the spin moments. With the decrease of the CNT curvatures from (3, 0) CNT to (10, 0) CNT, the spin magnetic moment decreases from 3.43 to 3.02 ${\mu }_{B}$, approaching that of FeN_3_@graphene. These results suggest that the curvatures of the substrates anchoring Fe atoms can affect the spin polarization of Fe atoms, and the larger the curvature, the greater the degree of the spin polarization.

**Figure 2:**
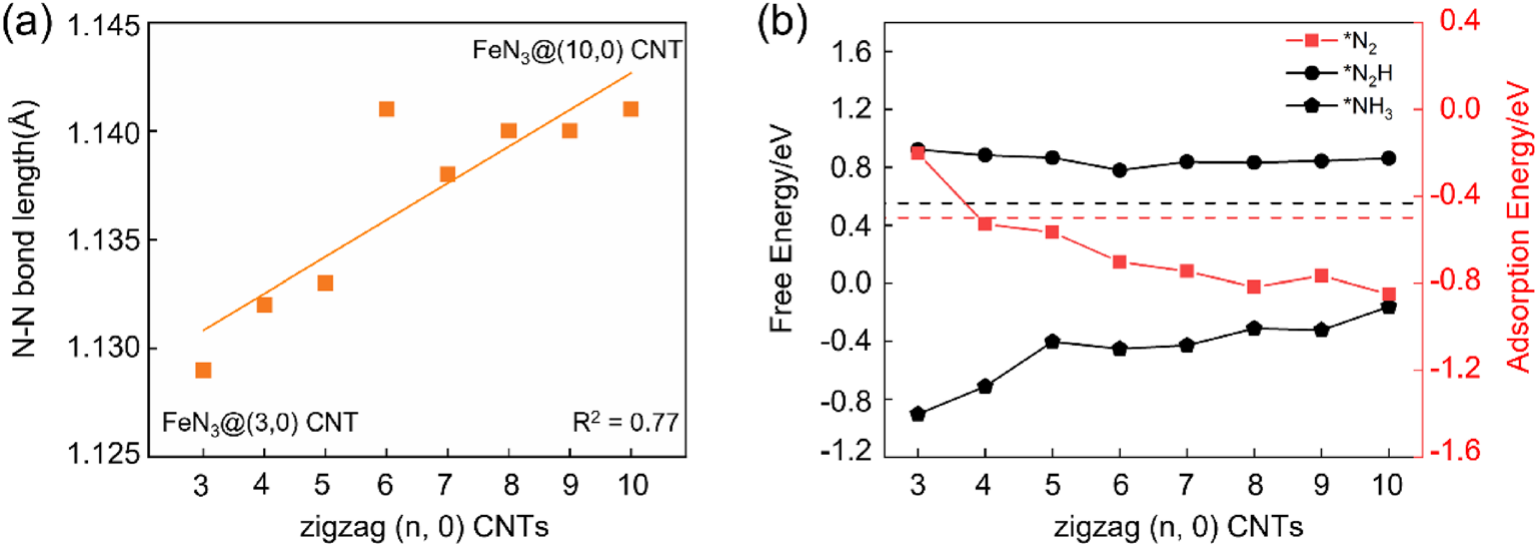
(**a**) The variation of N$\equiv$N bond lengths of N_2_ molecules adsorbed on FeN_3_@(n, 0) CNTs; (**b**) The adsorption energies of N_2_, the Gibbs free energy changes for the formation of *N_2_H and *NH_3_ in the N_2_ reduction reactions catalyzed by FeN_3_@(n, 0) CNTs (N_2_ adsorption mode is end-on).

Figure 2a demonstrates the variation of the N$\equiv$N bond lengths of N_2_ molecules adsorbed on FeN_3_@(n, 0) CNTs. There is a good linear relationship between the change of the N$\equiv$N bond lengths and the CNT curvatures, indicating that the CNT curvature plays an important role in weakening the N$\equiv$N bond. After adsorption, the N$\equiv$N bond is stretched from 1.10 Å in free N_2_ molecule to 1.129–1.141 Å, indicating that N_2_ is effectively activated by FeN_3_@(n, 0) CNTs. With the decrease of the CNT curvatures from (3, 0) CNT to (6, 0) CNT, the N$\equiv$N bond length increases from 1.129 to 1.141 Å. While the elongation of the N$\equiv$N bond by FeN_3_@(n, 0) CNTs (n = 7–10) are very similar, ranging from 1.138 to 1.141 Å. Therefore, reducing the curvature of CNTs can improve the activation of N_2_ molecules by FeN_3_ to a certain extent.

NRR process is complicated and can occur through distal, alternating, enzymatic and other mechanisms. During the NRR process, the effective adsorption of N_2_ on active sites of the catalyst and its activation are prerequisites. The chemisorption of N_2_ will make sufficient activation of the inert N≡N triple bond. According to previous studies, the formation of *N_2_H and *NH_3_ usually have relatively high free energies, thus making them the potential determining steps. Therefore, the following criteria have been proposed for the screening of eligible electrocatalysts for NRR^12^: (1) The adsorption energies of N_2_ should be lower than − 0.50 eV, corresponding to the chemisorption of N_2_ molecule, so as to ensure the effective activation of the inert N–N triple bonds; (2) The Gibbs free energy changes in the conversion processes of *N_2_ to *N_2_H (${\mathrm{\Delta G}}_{{\mathrm{N}}_{2}-{\mathrm{N}}_{2}\mathrm{H}}$) and *NH_2_ to *NH_3_ (${\mathrm{\Delta G}}_{{\mathrm{NH}}_{2}-{\mathrm{NH}}_{3}}$) should be lower than 0.55 eV to achieve an onset potential comparable to or lower than the predicted onset potential of the most efficient catalysts made of pure transition metals^44^. Therefore, in the following study, these three key steps in the N_2_ reduction reactions catalyzed by FeN_3_@(n, 0) CNTs, rather than all the reaction steps, were calculated to investigate the effect of the CNT curvatures on the NRR catalytic performance of FeN_3_@(n, 0) CNTs.

In Fig. 2b, the obtained adsorption energies of N_2_ on FeN_3_@(n, 0) CNTs, the Gibbs free energy changes ${\mathrm{\Delta G}}_{{\mathrm{N}}_{2}-{\mathrm{N}}_{2}\mathrm{H}}$ and ${\mathrm{\Delta G}}_{{\mathrm{NH}}_{2}-{\mathrm{NH}}_{3}}$ are shown. The N_2_ molecule can be adsorbed stably on all the FeN_3_@(n, 0) CNTs structures, but only the adsorption energy on the smallest (3, 0) CNT is higher than − 0.50 eV. With the decrease of the curvatures from (3, 0) to (10, 0) CNT, the adsorption of N_2_ becomes more stable. From (8, 0) to (10, 0) CNT, the adsorption energies vary in a very small range. This is because as the CNT diameter increases, the curvature of CNTs decreases, and their surfaces gradually become a plane. Therefore, the adsorption energy will gradually approach a limit, that is, the adsorption energy of N_2_ on FeN_3_@graphene. These results demonstrate that, except for CNTs with very small diameters, most other CNTs-based catalysts can adsorb N_2_ stably to ensure the effective activation of the inert N–N triple bonds.

For the formation of *N_2_H, the effect of CNT curvatures is very small. ${\mathrm{\Delta G}}_{{\mathrm{N}}_{2}-{\mathrm{N}}_{2}\mathrm{H}}$ varies in a very small range of 0.78–0.92 eV, indicating that all the FeN_3_@(n, 0) CNTs are inefficient for the catalysis of NRR. On the other hand, the CNT curvatures have a significant effect on the final protonation step of forming the *NH_3_ intermediate. From (3, 0) to (5, 0) CNT, ${\mathrm{\Delta G}}_{{\mathrm{NH}}_{2}-{\mathrm{NH}}_{3}}$ increases sharply from − 0.90 to − 0.40 eV. While, from (5, 0) to (10, 0) CNT, ${\mathrm{\Delta G}}_{{\mathrm{NH}}_{2}-{\mathrm{NH}}_{3}}$ increases much slowly from − 0.40 to − 0.16 eV. This shows that the decrease of the CNT curvatures will increase the Gibbs free energy barrier of the formation of the *NH_3_ intermediate, thus reducing the performance of the CNTs-based NRR catalyst.

After carefully examining the influence of CNT curvatures on the spin polarization of the Fe centers, the activation of the adsorbed N_2_ molecules, and three critical steps, we have selected (8, 0) CNT as the optimal substrate to anchor TM atoms to construct the computational models. Figure 1a shows the optimized structure of FeN_3_@(8, 0) CNT. The bond length between the Fe atom and the upper N atom is 1.84 Å, and the bond lengths of the other two Fe–N bonds are 1.95 Å. Owing to the relatively compact N_3_ pores, the adsorption site of Fe atom is slightly elevated above the plane composed of N atoms.

### Screening of TM atoms

**Figure 3:**
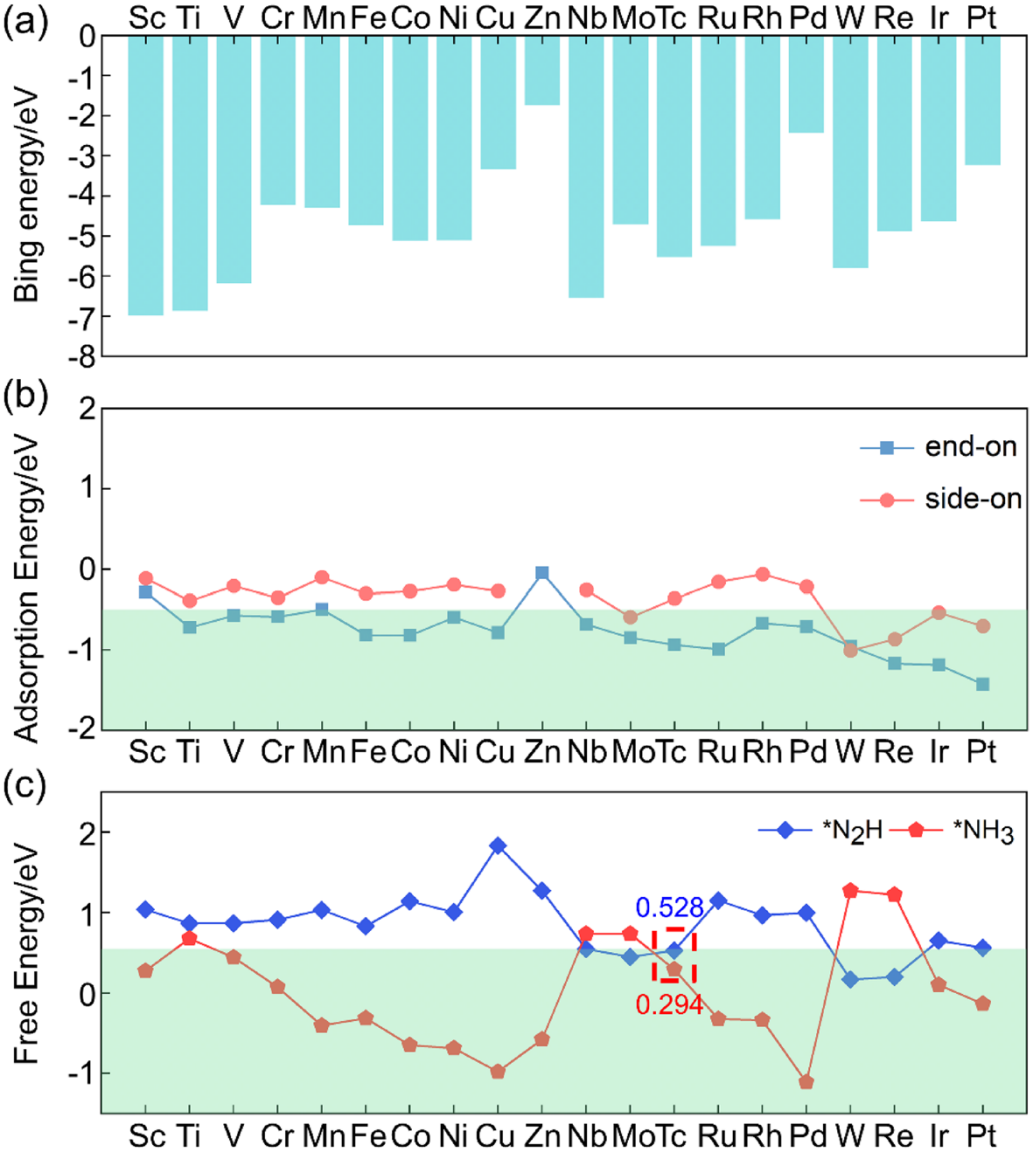
(**a**) The binding energies of 20 TM atoms on the surface of (8, 0) CNT; (**b**) The adsorption energies of N_2_ molecule on TMN_3_@(8, 0) CNTs via end-on and side-on configurations; (**c**) The Gibbs free energy changes in the formation of *N_2_H and *NH_3_ species catalyzed by TMN_3_@(8, 0) CNT. The numbers indicate ${\mathrm{\Delta G}}_{{\mathrm{N}}_{2}-{\mathrm{N}}_{2}\mathrm{H}}$ and ${\mathrm{\Delta G}}_{{\mathrm{NH}}_{2}-{\mathrm{NH}}_{3}}$ catalyzed by TcN_3_@(8, 0) CNT.

Next, 20 TM atoms (TM = Sc, Ti, V, Cr, Mn, Fe, Co, Ni, Cu, Zn, Nb, Mo, Tc, Ru, Rh, Pd, W, Re, Ir and Pt) anchored at the N_3_ center of (8, 0) CNT were screened to obtain an electrochemical NRR catalyst with excellent performance. The relative stability of TMN_3_@(8, 0) CNTs were evaluated by calculating the binding energies (E_b_) using the following equation:

3 $${\text{E}}_{{\text{b}}} = {\text{ E}}_{{{\text{TMN3}}@{\text{CNT}}}} {-}{\text{ E}}_{{{\text{CNT}}}} {-}{\text{ E}}_{{{\text{TM}}}} ,$$

where E_TM@CNT_, E_CNT_ and E_TM_ represent the energies of TMN_3_@(8, 0) CNTs, the (8, 0) CNT, and the isolated single TM atoms, respectively. As shown in Fig. 3a and Supplementary Table S1, all the calculated binding energies are negative, indicating that all the 20 TM atoms can be stably adsorbed by (8, 0) CNT. The binding energy of Zn atom is the weakest, while that of Sc atom is the strongest.

Following the above-mentioned criteria, the catalytic performance of TMN_3_@(8, 0) CNTs was then investigated. The adsorption energies of N_2_ on the TM atoms in both end-on and side-on fashions are shown in Fig. 3b and Supplementary Table S1. The results show that the end-on configuration is energetically more favorable than the side-on configuration for all TM atoms except for W atom. The capture of N_2_ via side-on configuration can not meet criterion 1 except for Mo, W, Re, Ir, and Pt. While, in the end-on configurations, only Sc, Mn and Zn are eliminated because of the higher adsorption energies. Therefore, the end-on configuration for N_2_ adsorption is employed to investigate the Gibbs free energy changes of the formation of *N_2_H and *NH_3_, and the results are shown in Fig. 3c and Supplementary Table S1. For the formation of *N_2_H, most of the TM atoms are ruled out as NRR catalysts because of the large Gibbs free energy change except for Nb, Mo, Tc, W, and Re. Further considering the formation of *NH_3_, Nb, Mo, W, and Re are ruled out. Eventually, TcN_3_@(8, 0) CNT is the only high-performance NRR candidate catalyst that meets all the above criteria.

Moreover, ab initio molecular dynamics (AIMD) simulations using canonical (NVT) ensemble is used to investigate the thermal stability of TcN_3_@(8, 0) CNT structure. After heating at the temperature of 500 K for 5 ps with a time step of 1 fs, we found that the structural reconstruction did not take place, implying that the TcN_3_@(8, 0) CNT structure can withstand temperature as high as 500 K. The variations of energy with respected to the time for AIMD simulations, and the snapshots of initial and final atomic configurations during the AIMD simulations are shown in Supplementary Fig. S1.

### NRR catalyzed by TcN_3_@(8, 0) CNT

**Figure 4:**
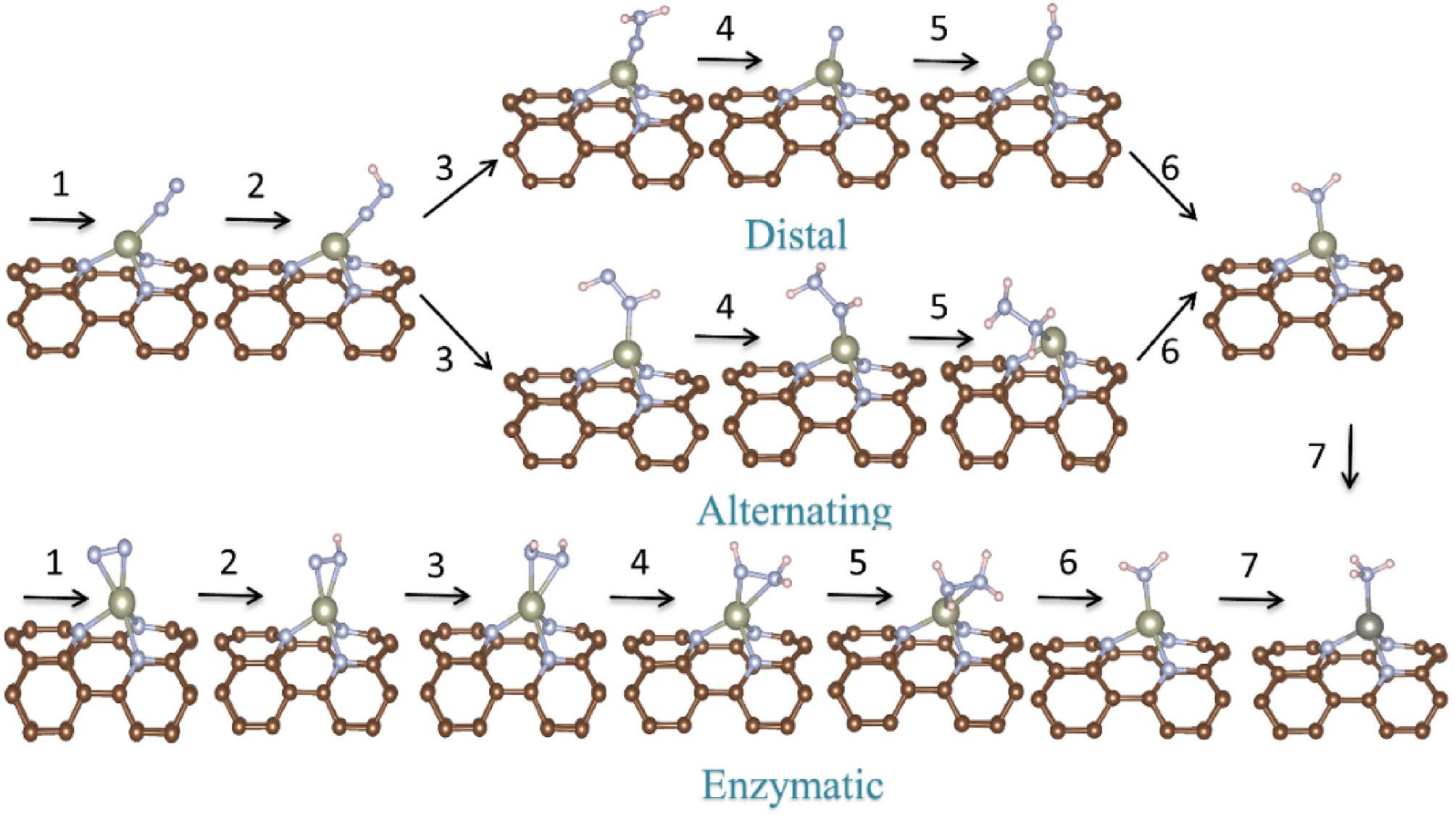
Schematic diagrams and optimized structures of the intermediates in distal, alternating, and enzymatic mechanisms for NRR catalyzed by TcN_3_@(8, 0) CNT.

The full NRR processes catalyzed by TcN_3_@(8, 0) CNT is further investigated via three possible pathways, including distal, alternating and enzymatic mechanisms. The schematic diagrams and the optimized structures of the intermediates in the three mechanisms are depicted in Fig. 4. The full reaction processes can be divided into seven steps, including the first step of N_2_ adsorption and six consecutive protonation steps. The distal and alternating mechanisms starts with the N_2_ end-on adsorption. In the distal mechanism, the distal N atom in the adsorbed *N_2_ will be fully hydrogenated via accepting three proton-electron pairs until the first NH_3_ molecule is released, then the proximal N atom continues to accept three proton-electron pairs to form the second NH_3_ molecule to complete the whole catalytic process. In the alternating mechanism, the remote and the proximal N atoms of the adsorbed *N_2_ alternately accept the proton–electron pairs to form NH_3_ molecules. The enzymatic mechanism starts with the N_2_ side-on configuration, and the six proton–electron pairs will be attached alternately to the two N atoms as in the alternating mechanism.

**Figure 5:**
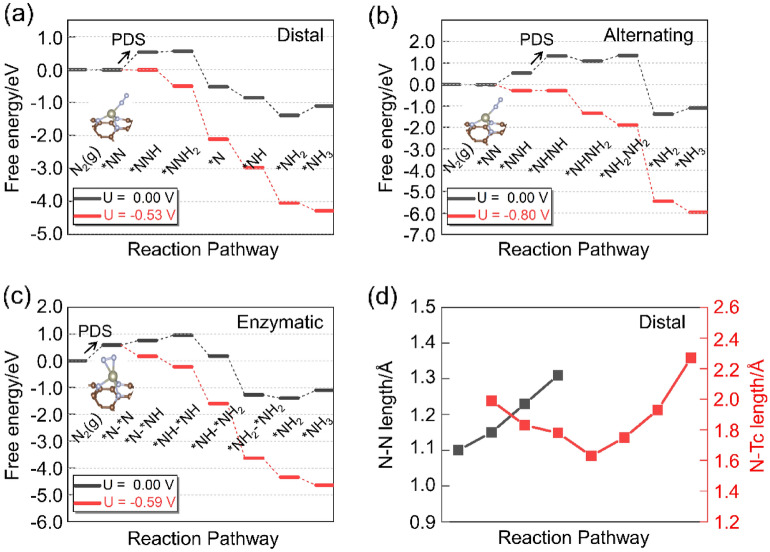
Gibbs free energy profiles for NRR via (**a**) distal; (**b**) alternating; (**c**) enzymatic pathways at different applied potentials. PDS is labeled in each Gibbs free energy profile. In the symbols depicting the intermediates, the N atom with “*” on its left means that it is adsorbed on the metal atom of the catalyst, and “N_x_H_y_” represents the formula of the intermediate. The illustrations show the structures of N_2_ molecules adsorbed on the catalyst surface. (**d**) The variation of N–N and Tc–N bond lengths via distal pathway.

Figure 5a–c are the calculated Gibbs free energy diagrams for the three possible NRR mechanisms. And the corresponding Gibbs free energy barriers are list in Supplementary Table S2. Our results show that the ΔG values of N_2_ adsorption via end-on and side-on fashions are − 0.01 eV and 0.59 eV, respectively. Compared with the side-on adsorption configuration, the end-on adsorption is more advantageous in terms of energy. Due to the extremely stable N–N triple bond in N_2_ molecule, breaking it to achieve protonation is often accompanied by a certain energy barrier. From the end-on configuration, the first protonation step requires an energy input of 0.53 eV. Then, the following protonation can take place through two paths, namely, the distal and alternating pathways. In the distal pathway, the energy consumption of the second protonation step (formation of the *N_2_H_2_ intermediate) is 0.03 eV. In the subsequent steps, the release of the first NH_3_ molecule and the first two protonation steps of the *N intermediate are all exothermal reactions. The formation of the *NH_3_ intermediate requires a 0.29 eV barrier in the last protonation step. Therefore, the maximum energy barrier of the whole distal reaction pathway is 0.53 eV and the first protonation step (formation of the *N_2_H intermediate) is the potential decisive step (PDS).

When the NRR occurs via the alternating pathway (Fig. 5b), the formation of the *N_2_H_2_ and *NH_2_NH_2_ intermediates are endothermal reactions and they require energy inputs of 0.80 and 0.26 eV, respectively. The second protonation step (*N_2_H → *N_2_H_2_) becomes the PDS for the alternating mechanism. In the enzymatic path, the maximum energy barrier of the six protonation steps is only 0.29 eV, and the N_2_ adsorption (0.59 eV) is the PDS. Here, we use the onset potential (U, its value is defined as: U = − ΔG_max_/e, where ΔG_max_ is the free energy variation of the PDS in each pathway) as a measure of the NRR performance. The onset potentials of the three possible mechanisms are − 0.53 V, − 0.80 V and − 0.59 V, respectively. Therefore, the distal mechanism is the most energetically favorable pathway for N_2_ reduction to ammonia catalyzed by TcN_3_@(8, 0) CNT. Previously, Tc atom has been reported to be the active center of catalyst for NRR. For example, tetracyanoquinodimethane molecules-supported Tc atom (Tc-rTCNQ) can catalyze NRR with a limiting potential of − 0.65 V^45^, while the limiting potential of Tc@N_6_-Graphene is − 0.56 V^46^. It can be observed that the utilization of suitable CNT as a substrate to adsorb Tc atom can effectively improve its catalytic performance.

Figure 5d shows the variation of N–N and Tc–N bond lengths in each step of the distal pathway. The N–N bond is firstly stretched from 1.10 Å in free N_2_ molecule to 1.15 Å in the *N_2_ intermediate, indicating that N_2_ is effectively activated by the adsorption on TcN_3_@(8, 0) CNT. Then the N–N bond is gradually elongated by protonation and finally breaks at the third protonation step. The Tc–N bond length is strongly correlated with the release of the first NH_3_ molecule. Before the formation of the *N intermediate, the Tc–N bond length decrease stepwise. And after that, the Tc–N bond length increases gradually until the release the second NH_3_ molecule.

**Figure 6:**
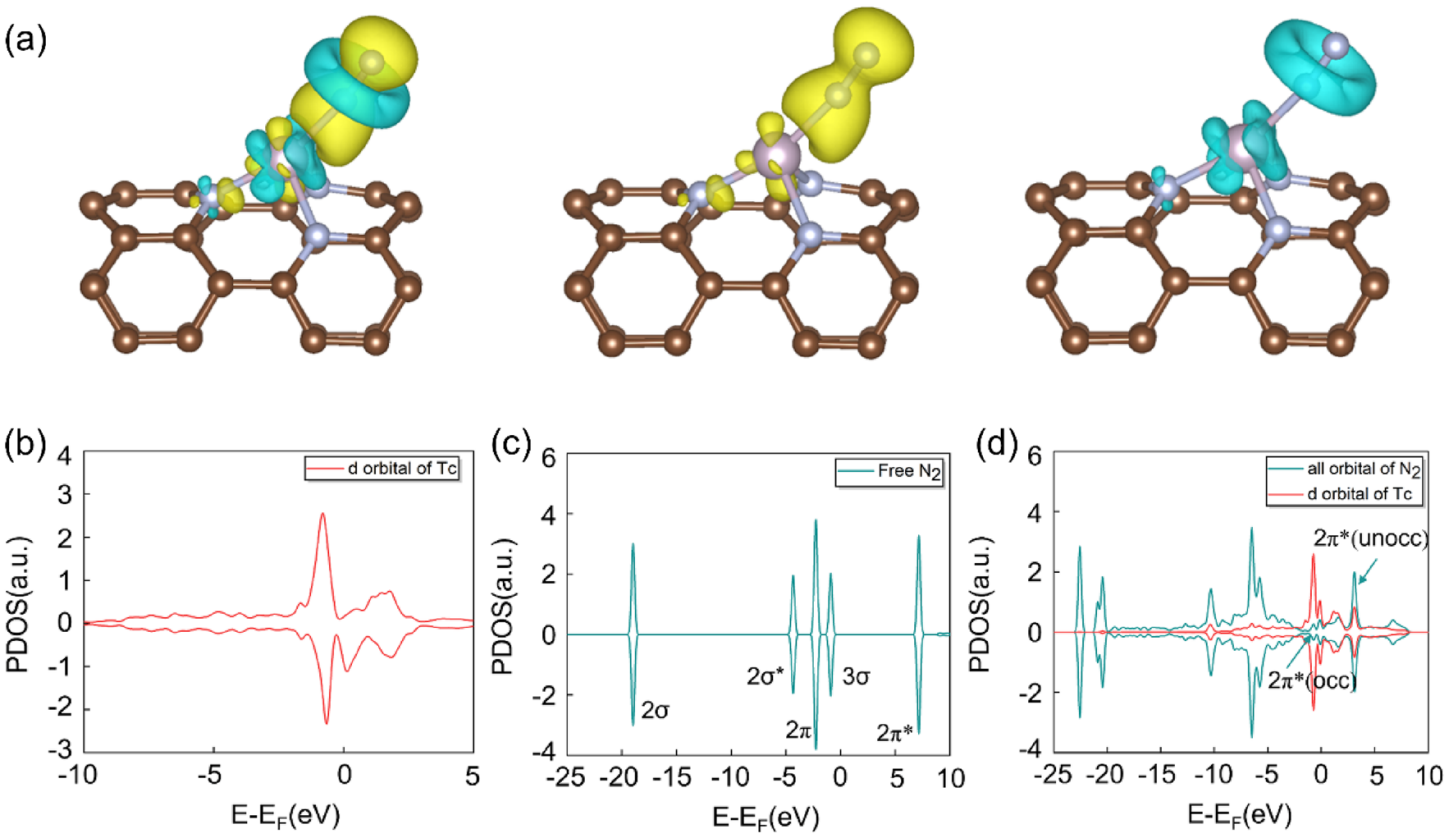
(**a**) The charge density difference of N_2_ adsorbed on TcN_3_@(8, 0) CNT in end-on configuration. The charge accumulation and depletion are depicted by yellow and cyan, respectively. The isosurface value is set as 0.005e/Å^3^. (**b**) The density of states of *d* orbitals of Tc before N_2_ is adsorbed on TcN_3_@(8, 0) CNT. (**c**) The projected density of states of free N_2_ molecule; (**d**) The density of states of N_2_ and the *d* orbitals of Tc atom after N_2_ is adsorbed on TcN_3_@(8, 0) CNT in the distal mechanism.

We further explore the charge density difference (Fig. 6a) and the variation of the projected density of states (PDOS) of N_2_ before and after adsorption in the distal mechanism (Fig. 6b–d). As shown in Fig. 6a, the results of charge density difference indicate that N_2_ molecule can interact with the catalyst and the charge transfer is obvious when adsorption occurs. The charges mainly accumulate on the N atoms, while the charges between the N–N is dissipated, indicating that the N_2_ molecule is activated and the strength of N–N bond is weakened. The charge accumulation and depletion between TcN_3_@(8, 0) CNT and N_2_ can be explained via the donation-back donation mechanism, in which the unoccupied *d* orbitals of Tc can accept electrons from the occupied orbitals of N_2_, simultaneously the *d* orbitals electrons of Tc can be transferred to the antibonding orbitals of N_2_. This can be confirmed by the PDOS of N_2_ molecule before and after adsorption on TcN_3_@(8, 0) CNT as shown in Fig. 6b–d. The degenerate 2π orbital and 2π* orbital in free N_2_ molecule split into individual occupied orbitals after the adsorption of N_2_ on the TcN_3_@(8, 0) CNT surface. Some electrons are transferred from the 3σ and spilt 2π orbitals to the unoccupied *d* orbitals of Tc atom, which can effectively enhance the adsorption of N_2_ molecule. Meanwhile, the 2π* antibonding orbitals split into two parts, i.e., occupied and unoccupied orbitals, in which the electrons of the occupied orbitals originate from the back donation of the occupied *d* orbitals of Tc. The strong *d*-2π* interaction between N_2_ molecule and Tc atom is the key to promote the activation of the N_2_ molecule. Therefore, the adsorbed N_2_ on TcN_3_@(8, 0) CNT surface can be activated efficiently, which also explains why TcN_3_@(8, 0) CNT can efficiently catalyze the N_2_ reduction reaction via distal mechanism.

### Charge variation in the distal pathway and HER competition

**Figure 7:**
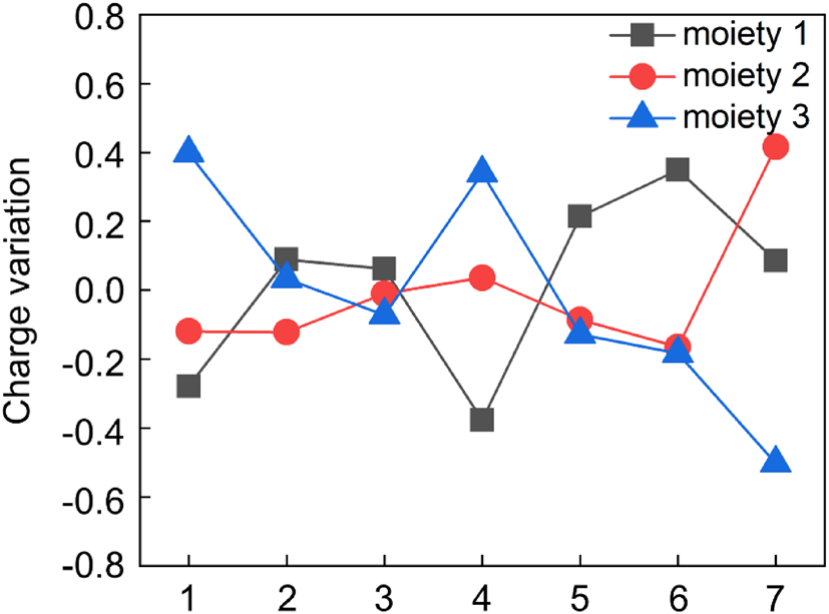
Bader charge variation of the three moieties along the distal mechanism. Moieties 1, 2 and 3 represent (8, 0) CNT, TcN_3_, and the adsorbed N_x_H_y_ species, respectively. The abscissa represents reaction steps in the distal mechanism, as depicted in Fig. 4.

In order to further understand the superior NRR catalytic performance of TcN_3_@(8, 0) CNT, we calculated the Bader charge variation of the reaction intermediates in the favorable distal mechanism and the results are shown in Fig. 7 and Supplementary Table S3. According to previous studies^47–49^, we divided each intermediate in this process into three parts: moiety 1 (carbon nanotube), moiety 2 (TcN_3_), and moiety 3 (the adsorbed N_x_H_y_ species). The charge variation refers to the charge difference of each moiety between the current step and the previous step. The charge variation at the first step demonstrates that the N_2_ molecule obtains 0.40*e* from CNT and TcN_3_ moieties during the end-on fashion adsorption. Both CNT and TcN_3_ play an important role in the activation process of the adsorbed N_2_ molecule. TcN_3_ donates electrons to both CNT and moiety 3 at the second step. At the third and fourth steps, the charge variation of TcN_3_ is about zero, and the charge variation of moiety 1 and moiety 3 is complementary to each other. While TcN_3_ and moiety 3 get almost the same amount of electrons from CNT at the fifth and the sixth step. At the final step, the second NH_3_ molecule is formed and moiety 3 returns the excess electrons to the catalyst itself. Based on the above analysis, we found that CNT acts as an electron reservoir in the whole catalytic process, while the active center TcN_3_ acts sometimes as a bridge to transport electrons. Both these two parts make an important contribution to the high NRR catalytic performance of TcN_3_@(8, 0) CNT.

In addition to the structural stability and high catalytic activity, an idea NRR catalyst should be also able to suppress the HER, a key side reaction in NRR, to achieve the high Faraday efficiency (FE). Therefore, we evaluated the catalytic selectivity of TcN_3_@(8, 0) CNT via two measures. On the one hand, the adsorption energies of N_2_ and proton on the catalyst are calculated to be − 0.94 eV and − 0.81 eV, respectively. The adsorption energy results indicate that the adsorption of N_2_ molecule is more stable than that of proton, which prevents the adsorption of a large number of protons on the catalyst surface, thus hindering the HER process. On the other hand, the measure of the difference of the limiting potentials between NRR and HER is calculated as: ΔU = U_PDS_(NRR)—U_PDS_(HER), where U_PDS_(NRR) and U_PDS_(HER) respectively represent the limiting potentials of NRR and HER^16,48^. A positive value represents that HER is suppressed to enhance the selectivity of NRR^50^. Our calculation results show that the limit potential of HER is − 0.62 V. Hence, ΔU is 0.09 V (As shown in Supplementary Fig. S2), which demonstrates its high selectivity to NRR.

## Conclusion

In summary, first-principles calculations were performed to explore high efficiency NRR single-atom catalysts based on CNTs. At first, the effect of CNT diameters on the NRR catalytic performance of CNTs based catalysts were investigated. The results showed that CNT curvatures have a significant effect on the spin polarization of the catalytic active centers, the activation of the adsorbed N_2_ molecules and the Gibbs free energy barriers for the formation of the *NH_3_ intermediate, but have little effect on the formation of *N_2_H intermediate. With the decrease of the CNT curvatures, the adsorption of N_2_ will become more effective, but the onset potential of the formation of the *NH_3_ intermediate will also increase, thus reducing the performance of the CNTs-based NRR catalyst. Therefore, zigzag (8, 0) CNT was selected as the substrate to anchor twenty TM atoms (Sc, Ti, V, Cr, Mn, Fe, Co, Ni, Cu, Zn, Nb, Mo, Tc, Ru, Rh, Pd, W, Re, Ir, and Pt) and their catalytic ability were investigated systematically according to the criteria for the screening of eligible electrocatalysts for NRR. TcN_3_@(8, 0) CNT is the only possible candidate catalyst for high performance NRR after screening, and our calculations illustrate that NRR prefers the distal pathway with a low limiting potential of − 0.53 V. The strong *d*-2π* interaction between the active center and N_2_ molecule is the key to facilitate the N_2_ molecule activation. Furthermore, TcN_3_@(8, 0) CNT exhibits higher selectivity for NRR than the competing HER process. Considering that Tc is a radioactive material, in the future, a pseudo-Tc material^51^ (such as Mo–Ru alloy) may be designed based on the concept of density of states engineering to effectively prevent any potential risks associated with radioactivity while retaining the desirable catalytic properties. We expect that our results would inspire more research on low-dimensional carbonaceous materials in the field of NRR electrocatalysis.

## Supplementary Information

Supplementary Information.

## Data Availability

Data are available from the corresponding author on reasonable request.
