# Supplementary material for: High efficiency carbon nanotubes-based single-atom catalysts for nitrogen reduction
Source: Sci Rep. 2023 Jun 19;13:9926. doi: 10.1038/s41598-023-36945-0 (PMC10279692; doi:10.1038/s41598-023-36945-0)
Supplement: Supplementary file 1 — Supplementary Information. [file 41598_2023_36945_MOESM1_ESM.docx]

**High efficiency carbon nanotubes-based single-atom catalysts for nitrogen reduction**

*Wei Liu^a,†^,* *Kai Guo^a,†^, Yunhao Xie^a^, Sitong Liu^a^, Liang Chen^b,a^, and Jing Xu^a,*^*

^a^College of Optical, Mechanical and Electrical Engineering, Zhejiang A&F University, Hangzhou, Zhejiang, 311300, P.R. China

^b^School of Physical Science and Technology, Ningbo University, Ningbo, Zhejiang, 315211, P.R. China

^†^These authors contributed equally to this work.

*Correspondence to: jingxu@zafu.edu.cn

**Table S1** The binding energies (E_b_) of 20 transition metal atoms on N_3_@(8,0) CNT; the adsorption energies of N_2_ molecule on TMN_3_@(8, 0) CNTs via end-on and side-on configurations; the Gibbs free energy changes in the formation of *N_2_H (${\Delta G}_{N_{2}-N_{2}H}$) and *NH_3_ (${\Delta G}_{{NH}_{2}-\mathrm{NH}_{3}}$) species catalyzed by TMN_3_@(8, 0) CNT.

| **System** | **E_b_ (eV)** | **Adsorption Energy (eV)** | | $\boldsymbol{\Delta G}_{\mathbf{N}_{\mathbf{2}}\mathbf{-}\mathbf{N}_{\mathbf{2}}\mathbf{H}}$**(eV)** | $\boldsymbol{\Delta G}_{{\mathbf{N}\mathbf{H}}_{\mathbf{2}}\mathbf{-}\mathbf{NH}_{\mathbf{3}}}$ **(eV)** |
| --- | --- | --- | --- | --- | --- |
|  |  | End-on | Side-on |  |  |
| ScN_3_@(8, 0) CNT | -6.978 | -0.285 | -0.115 | 1.040 | 0.274 |
| TiN_3_@(8, 0) CNT | -6.868 | -0.723 | -0.392 | 0.865 | 0.679 |
| VN_3_@(8, 0) CNT | -6.180 | -0.575 | -0.210 | 0.868 | 0.445 |
| CrN_3_@(8, 0) CNT | -4.223 | -0.590 | -0.355 | 0.913 | 0.074 |
| MnN_3_@(8, 0) CNT | -4.299 | -0.499 | -0.102 | 1.034 | -0.404 |
| FeN_3_@(8, 0) CNT | -4.738 | -0.818 | -0.304 | 0.832 | -0.312 |
| CoN_3_@(8, 0) CNT | -5.115 | -0.820 | -0.274 | 1.144 | -0.647 |
| NiN_3_@(8, 0) CNT | -5.106 | -0.597 | -0.193 | 1.007 | -0.686 |
| CuN_3_@(8, 0) CNT | -3.334 | -0.789 | -0.271 | 1.835 | -0.979 |
| ZnN_3_@(8, 0) CNT | -1.745 | -0.047 | -- | 1.273 | -0.576 |
| NbN_3_@(8, 0) CNT | -6.534 | -0.686 | -0.258 | 0.549 | 0.738 |
| MoN_3_@(8, 0) CNT | -4.702 | -0.852 | -0.596 | 0.449 | 0.738 |
| TcN_3_@(8, 0) CNT | -5.521 | -0.938 | -0.366 | 0.528 | 0.294 |
| RuN_3_@(8, 0) CNT | -5.250 | -0.995 | -0.159 | 1.152 | -0.323 |
| RhN_3_@(8, 0) CNT | -4.574 | -0.673 | -0.063 | 0.966 | -0.336 |
| PdN_3_@(8, 0) CNT | -2.426 | -0.713 | -0.217 | 1.000 | -1.108 |
| WN_3_@(8, 0) CNT | -5.795 | -0.957 | -1.010 | 0.168 | 1.274 |
| ReN_3_@(8, 0) CNT | -4.876 | -1.172 | -0.868 | 0.200 | 1.223 |
| IrN_3_@(8, 0) CNT | -4.638 | -1.189 | -0.539 | 0.653 | 0.102 |
| PtN_3_@(8, 0) CNT | -3.223 | -1.429 | -0.707 | 0.563 | -0.134 |

**Table S2** The Gibbs free energy barriers for NRR catalyzed by TcN_3_@ (8,0) CNT.

| **Distal** | | **Alternating** | | **Enzymatic** | |
| --- | --- | --- | --- | --- | --- |
| Adsorbed  species | Free Gibbs  Energy(eV) | Adsorbed  species | Free Gibbs  Energy(eV) | Adsorbed  species | Free Gibbs  Energy(eV) |
| *NN | -0.01 | *NN | -0.01 | *N-*N | 0.59 |
| *NNH | 0.52 | *NNH | 0.52 | *N-*NH | 0.76 |
| *NNH_2_ | 0.56 | *NHNH | 1.32 | *NH-*NH | 0.96 |
| *N | -0.52 | *NHNH_2_ | 1.09 | *NH-*NH_2_ | 0.18 |
| *NH | -0.85 | *NH_2_NH_2_ | 1.35 | *NH_2_-*NH_2_ | -1.27 |
| *NH_2_ | -1.39 | *NH_2_ | -1.39 | *NH_2_ | -1.39 |
| *NH_3_ | -1.10 | *NH_3_ | -1.10 | *NH_3_ | -1.10 |

**Table S3** Bader charge variation of the three moieties along the distal mechanism catalyzed by TcN_3_@(8, 0) CNT. Moieties 1, 2 and 3 represent (8, 0) CNT, TcN_3_, and the adsorbed N_x_H_y_ species, respectively.

| **Distal Mechanism** | | | |
| --- | --- | --- | --- |
| Adsorbed species | Moiety1 | Moiety2 | Moiety3 |
| *NN | -0.28 | -0.12 | 0.40 |
| *NNH | 0.09 | -0.12 | 0.03 |
| *NNH_2_ | 0.06 | -0.01 | -0.07 |
| *N | -0.38 | 0.04 | 0.34 |
| *NH | 0.21 | -0.09 | -0.13 |
| *NH_2_ | 0.35 | -0.02 | -0.18 |
| *NH_3_ | 0.09 | 0.42 | -0.50 |

**
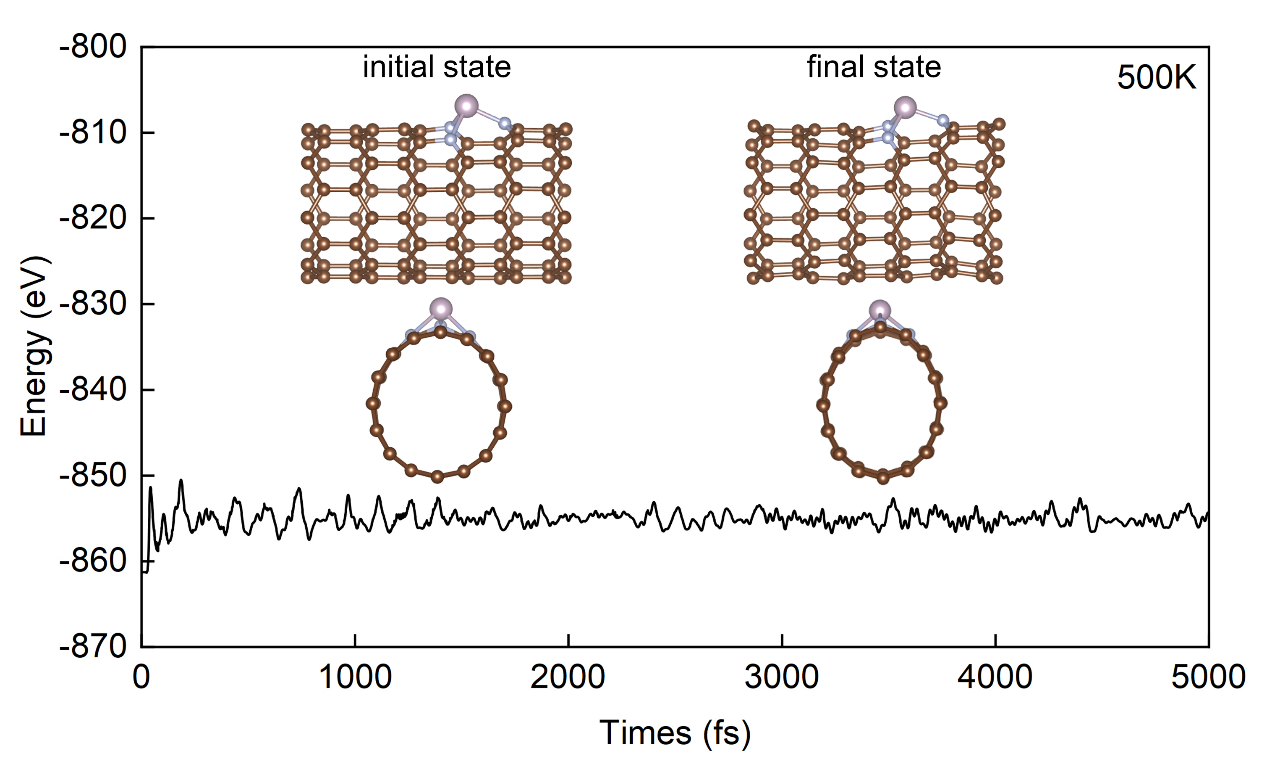
**

**Figure S1.** The variations of energy with respected to the time for AIMD simulations of TcN_3_@(8, 0) CNT under 500 K with a time step of 1 fs. The snapshots of the initial and final atomic configurations during the AIMD simulations.





**Figure S2.** Limiting potentials for NRR UL(NH_3_), HER UL(H_2_) and the difference between them UL(NH_3_) - UL(H_2_) on the TcN_3_@(8, 0) CNT.
